# Overexpression of Mitochondrial Catalase within Adipose Tissue Does Not Confer Systemic Metabolic Protection against Diet-Induced Obesity

**DOI:** 10.3390/antiox12051137

**Published:** 2023-05-22

**Authors:** Amanda J. Croft, Conagh Kelly, Dongqing Chen, Tatt Jhong Haw, Aaron L. Sverdlov, Doan T. M. Ngo

**Affiliations:** 1 School of Medicine and Public Health, University of Newcastle, Callaghan, NSW 2308, Australia; 2 Hunter Medical Research Institute, New Lambton Heights, NSW 2305, Australia; 3 School of Biomedical Sciences and Pharmacy, University of Newcastle, Callaghan, NSW 2308, Australia; 4 Hunter New England Local Health District, Newcastle, NSW 2267, Australia

**Keywords:** obesity, adipose tissue, mitochondrial catalase, oxidative stress, mitochondria, insulin resistance, metabolism

## Abstract

Obesity is associated with significant metabolic co-morbidities, such as diabetes, hypertension, and dyslipidaemia, as well as a range of cardiovascular diseases, all of which lead to increased hospitalisations, morbidity, and mortality. Adipose tissue dysfunction caused by chronic nutrient stress can result in oxidative stress, mitochondrial dysfunction, inflammation, hypoxia, and insulin resistance. Thus, we hypothesised that reducing adipose tissue oxidative stress via adipose tissue-targeted overexpression of the antioxidant mitochondrial catalase (mCAT) may improve systemic metabolic function. We crossed mCAT (floxed) and Adipoq-Cre mice to generate mice overexpressing catalase with a mitochondrial targeting sequence predominantly in adipose tissue, designated AdipoQ-mCAT. Under normal diet conditions, the AdipoQ-mCAT transgenic mice demonstrated increased weight gain, adipocyte remodelling, and metabolic dysfunction compared to the wild-type mice. Under obesogenic dietary conditions (16 weeks of high fat/high sucrose feeding), the AdipoQ-mCAT mice did not result in incremental impairment of adipose structure and function but in fact, were protected from further metabolic impairment compared to the obese wild-type mice. While AdipoQ-mCAT overexpression was unable to improve systemic metabolic function per se, our results highlight the critical role of physiological H_2_O_2_ signalling in metabolism and adipose tissue function.

## 1. Introduction

Obesity is recognised as a chronic relapsing condition that has emerged as a major public health problem. The World Health Organisation reported in 2016 that the number of adults globally who are overweight or obese is approximately 1.9 billion [1]. Obesity is associated with significant cardiovascular (CV) and metabolic (cardio-metabolic) co-morbidities, such as diabetes, hypertension, dyslipidaemia, coronary heart disease, heart failure, and atrial fibrillation, all of which are closely linked to increased hospitalisations, morbidity, and mortality [2,3,4].

Physiologically, adipose tissue has a remarkable ability to rapidly expand in times of nutrient surplus by increasing both the adipocyte size and number, accompanied by increased vascularisation [5,6]. However, chronic nutrient excess, as occurs in obesity, can result in the excessive expansion of adipose tissue, which leads to its dysfunction and is associated with increased inflammation, reduced vascularisation, hypoxia, mitochondrial dysfunction, oxidative stress, changes to the adipokine secretome, and insulin resistance [7,8]. It has been suggested that metabolic regulation is largely dependent on mitochondria, which play an important role in energy homeostasis, nutrient metabolism, ATP, and heat production [9]. The excess production of mitochondrial reactive oxygen species (ROS), including superoxide (O_2_^−^), hydrogen peroxide (H_2_O_2_), and the hydroxyl radical (OH), and decreased activity of antioxidant defences, such as catalase, glutathione peroxidases, and peroxiredoxins, have been postulated to contribute to obesity-induced metabolic perturbations [10,11,12,13]. However, despite the abundant literature suggesting oxidative stress is a critical pathophysiological mechanism of obesity-induced metabolic dysfunction, strategies to reduce systemic oxidative stress using dietary supplementation with antioxidants, such as vitamins A, C, and E, had little effect on type-II diabetes risk [14,15], suggesting a more targeted approach may be required.

Mitochondrial dysfunction within adipose tissue, characterised by increased ROS production, has been linked to metabolic abnormality [16,17]. Mice overexpressing mitochondrial-targeted catalase (mCAT) have reduced mitochondrial ROS production compared with wild-type (WT) littermates, with associated improvements in the lifespan and improved cardiac and systemic metabolic function under diet-induced obesogenic stress [18,19,20,21,22]. To date, it remains largely unknown whether targeting mCAT to visceral adipose tissue is an adequate strategy to mitigate systemic obesogenic stress-induced metabolic perturbations. In this study, we examined and described the effects of the overexpression of mCAT in adipose tissue on the systemic metabolic profile during diet-induced obesity.

## 2. Materials and Methods

### 2.1. Animals

To generate the adipose tissue-targeted mitochondrial catalase overexpression (AdipoQ-mCAT) mouse model, AdipoQ-Cre (B6;FVB-Tg(Adipoq-cre)1Evdr/J, stock no. 010803, Jackson Laboratories; Bar Harbor, ME, USA) mice were bred with floxed mCAT mice (B6N.B6-Tg(GAPDH-OTC/CAT)6424Prab/J, stock no. 030712, Jackson Laboratories). All the mice were on a C57BL/6NJ background. The offspring were genotyped by PCR analysis of DNA obtained from tail-snip biopsies using transgene-specific oligonucleotide primers for human mitochondrial catalase and AdipoQ genes. Age-matched wild-type C57BL/6NJ mice were used as controls. At 6–8 weeks of age, male and female mice were fed either (1) a standard chow diet (normal chow; NC) containing 4.6% fat/0.0% sucrose by weight, where 12.0% of the energy was calculated to be supplied from lipids or (2) a high fat, high sucrose (HFHS) diet containing 36.0% fat/34.6% sucrose by weight, where 59.0% of the energy was calculated to be supplied from lipids (SF03-002, Specialty Feeds, Western Australia). All the animals were maintained on their respective diets for 16 weeks. The animals were housed in groups of 4 per cage. The body weight was recorded weekly for the duration of the diet. All the animal experiments were approved by the University of Newcastle Animal Care and Ethics Committee, Approval Number A-2017-736.

### 2.2. RNA Isolation

RNA was isolated from visceral white adipose tissue (WAT), heart, liver, and skeletal muscle as follows: 1 mL of Trizol (Invitrogen 15596026; Waltham, MA, USA) was added to each tissue sample along with a stainless-steel bead. The tissues were disrupted with a TissueLyser II (QIAGEN; Hilden, Germany) in 30 s bursts until the tissue was adequately homogenised. The RNA was isolated from the lysate using an Isolate II RNA mini kit (Bioline BIO-52073; Cincinnati, OH, USA), according to the manufacturer’s protocol. The RNA was quantitated by spectrophotometry (NanoDrop 2000, ThermoFisher Scientific; Waltham, MA, USA), and the quality was assessed using TapeStation analysis of RNA integrity (Agilent Technologies; Santa Clara, CA, USA).

### 2.3. RT Profiler qPCR Arrays

Changes in gene expression were analysed using RT2 Profiler qPCR arrays (Mouse Oxidative Stress and Antioxidant Defense, QIAGEN PAMM-065Z; Hilden, Germany). The RNA was converted into cDNA using the RT2 First Strand Kit (QIAGEN 330404; Hilden, Germany), according to the manufacturer’s instructions. In brief, the RNA isolated from mouse WAT was pooled in equal quantities for each treatment group to achieve a final input of 500 ng RNA per cDNA reaction. The reactions were incubated at 42 °C for 15 min, then inactivated by incubating them at 95 °C for 5 min. qPCR of 84 mouse genes involved in oxidative stress response and 5 housekeeper genes was carried out according to the manufacturer’s instructions. Briefly, equal amounts of cDNA were added to RT2 SYBR Green/ROX qPCR master mix (QIAGEN 3300523; Hilden, Germany). qPCR thermal cycling was carried out using the Applied Biosystems QuantStudio 6 Flex System (Waltham, MA, USA), as follows: (10 min, 95 °C) × 1 cycle; (15 s, 95 °C; 60 s, 60 °C) × 40 cycles. The data were analysed using GeneGlobe Data Analysis Center (QIAGEN, Hilden, Germany). Gene expression heat maps were generated using Partek Genomics Suite (Midpoint Orchard, Singapore).

### 2.4. Quantitative PCR

cDNA was prepared by reverse transcription of 1 μg of total RNA using a High-Capacity cDNA Reverse Transcription kit according to the manufacturer’s protocol (Applied Biosystems, Waltham, MA, USA). Quantitative PCR was carried out using TaqMan or SYBR Green chemistry using gene-specific primers on an Applied Biosystems QuantStudio 6 Real-Time PCR System (Waltham, MA, USA). PPIA, GAPDH, or HSP90ab1 were used as internal controls. The primer details are recorded in Supplementary Tables S1 and S2.

### 2.5. Insulin Stimulation of Adipose Tissue

The freshly dissected WAT was cut into 1–2 mm pieces and incubated at 37 °C, 5% CO_2_ overnight in EGM-2 Endothelial Cell Growth Medium-2 (Lonza, CC-3162; Basel, Switzerland) without growth factors containing 0.5% foetal bovine serum (FBS), penicillin/streptomycin and amphotericin B (Sigma-Aldrich, A2942; St. Louis, MO, USA). The tissue was treated with a vehicle or 100 nM Humulin R insulin for 30 min at 37 °C, 5% CO_2_ before it was snap frozen and stored at −80 °C until analysis.

### 2.6. Protein Isolation

The WAT was homogenised by grinding with a cold mortar and pestle into ice-cold RIPA buffer (Cell Signaling Technologies, 9806; Danvers, MA, USA) containing protease and phosphatase inhibitors 1 and 3 (Sigma-Aldrich, P8340, P2850, and P0044; St. Louis, MO, USA). The tissue homogenates were kept on ice for 30 min before freeze-thawing 2 times using liquid nitrogen to assist the tissue lysis. The tissue debris was removed by centrifugation for 30 min at 12,000 rpm, 4 °C, and the cleared lysates were transferred to new tubes, avoiding the floating lipid layer. The total protein levels were quantitated by a DC protein assay (Bio-Rad 5000112; Hercules, CA, USA).

### 2.7. ELISA

The phosphorylated and total AKT levels were measured using the Akt (pS473) + total AKT ELISA kit (Abcam, ab126433; Cambridge, UK), according to the manufacturer’s instructions. Briefly, the WAT protein lysates were loaded at 20 μg total protein per well and incubated overnight at 4 °C. Primary anti-phospho-AKT (Ser473) or anti-pan-AKT antibodies were added, and the plates were incubated at room temperature for 1 h. HRP-conjugated secondary antibodies were incubated at room temperature for 1 h. TMB One-step substrate was incubated for 30 min before Stop Solution was added. The absorbance at 450 nm was measured for each plate. The expression levels were calculated as a ratio between the phosphorylated and total AKT levels from each sample and analysed using GraphPad Prism 9 software.

### 2.8. Catalase Activity Assay

The total catalase activity was measured in the WAT using the Catalase Colorimetric Activity Kit (Invitrogen, EIACATC; Waltham, MA, USA), according to the manufacturer’s protocol. Briefly, the WAT was homogenised in 0.5 mL cold 1× Assay Buffer per 100 mg of tissue using the TissueLyser II. The lysates were centrifuged at 10,000× *g* for 15 min at 4 °C. Avoiding the lipid layer, the cleared lysates were transferred to fresh tubes. The total protein content was quantitated using a DC protein assay, and the samples were normalised to 1.6 μg/mL with 1× Assay Buffer. The assay was then carried out according to the kit protocol. GraphPad Prism 9 software was used to generate the standard curve and interpolate the catalase activity values for the samples.

### 2.9. Glucose Tolerance Test

After 16 weeks of feeding on a normal chow or HFHS diet, the fasting glucose levels were measured using an Accuchek Guide glucometer. The mice were injected intraperitoneally with 50% glucose at a dose of 2 g/kg. Blood glucose readings were taken at 15, 30, 45, 60, 90, and 120 min post-injection. The glucose readings were plotted over time, and the area under the curve analysis was carried out using GraphPad Prism 9 software.

### 2.10. Insulin Tolerance Test

The mice were injected intraperitoneally with insulin at a dose of 0.6 U/kg. Blood glucose readings were taken at 15, 30, 45, 60, 90, and 120 min post-injection. The glucose readings were plotted over time and the area under the curve analysis was carried out using GraphPad Prism 9 software.

### 2.11. Triglyceride Assay

The triglyceride levels were measured in mouse plasma samples using the Triglyceride Colorimetric Assay kit (Cayman Chemical, 10,010,303; Ann Arbor, MI, USA), according to the manufacturer’s protocol. In brief, the plasma samples were diluted at a ratio of 1:2 in Standard Diluent and the standards were diluted according to the protocol. An enzyme mixture was added to the samples, which were incubated for 60 min at room temperature before the absorbance was measured at 540 nm. The triglyceride concentrations were calculated from the standard curve.

### 2.12. H & E Staining

Paraffin-embedded WAT sections (5 μM) were rehydrated with the following incubations: xylene, 2 × 3 min; 100% ethanol, 2 × 3 min; 95% ethanol, 3 min; 70% ethanol, 3 min; 50% ethanol, 3 min; dH_2_O, 3 min. Slides were stained with haematoxylin for 2 min with agitation before washing in tap water. The slides were incubated with Scott’s Tap Water Substitute until the staining was blue in colour. The slides were stained with Eosin Y solution until optimal staining was achieved, then washed in tap water. Rehydration was carried out with the following incubations: 100% ethanol, 2 × 3 min; xylene, 2 × 3 min. The slides were cover slipped with Ultramount No. 4 mounting medium. The adipocyte area was quantitated from at least five different randomly generated fields of view at 40× magnification using ImageJ/Adiposoft software [23]. At least 250 adipocytes were analysed per group.

### 2.13. Adipogenic Profiling

#### 2.13.1. Isolation of Bone Marrow Cells

Mouse bone marrow cells were isolated by flushing the femurs and tibias with High Glucose DMEM (Sigma-Aldrich, D5796; St. Louis, MO, USA) containing 15% FBS (Sigma-Aldrich, F9423; St. Louis, MO, USA) supplemented with penicillin/streptomycin (P/S) (Sigma-Aldrich, P4333; St. Louis, MO, USA) into 50 mL tubes capped with 70 μM cell strainers. The cells were pelleted by centrifugation at 1000 rpm for 5 min. The pellets were resuspended with Red Blood Cell Lysis Buffer (Sigma-Aldrich, Z642843; St. Louis, MO, USA) and incubated for 5 min at room temperature before being neutralised with 9 mL DMEM (15% FBS + P/S). The cells were pelleted by centrifugation at 1000 rpm, 5 min, before being resuspended in 9 mL DMEM (15% FBS + P/S) and aliquoted into a 6-well cell culture plate. The bone marrow cells were incubated at 37 °C, 5% CO_2_ for 3–4 days to allow attachment, at which time the media was replenished with fresh DMEM (15% FBS + P/S).

#### 2.13.2. Adipogenesis Assay

When the cultured bone marrow cells reached approximately 90% confluency, adipocyte differentiation was initiated (Day 0) by adding induction media consisting of High Glucose DMEM supplemented with 10% FBS, P/S, 2 μM Insulin (Sigma-Aldrich, I9278), 20 μM Rosiglitazone (Sigma-Aldrich, R2408; St. Louis, MO, USA), 500 μM 3-isobutyl-1-methylxanthine (Sigma-Aldrich, I7018; St. Louis, MO, USA), and 1 μM Dexamethasone (Sigma-Aldrich, D4902; St. Louis, MO, USA). On Day 2, the media was replaced with fresh induction media. On Day 4, the media was changed to base media consisting of High Glucose DMEM supplemented with 10% FBS, P/S, 2 μM Insulin (Sigma-Aldrich, I9278; St. Louis, MO, USA), and 20 μM Rosiglitazone (Sigma-Aldrich, R2408; St. Louis, MO, USA). On Day 7, the differentiated cells were stained with Oil Red O. The amount of Oil Red O was quantified by destaining the cells with isopropanol and measuring the absorbance at 490 nm. The Oil Red O content was determined by interpolation from a standard curve of known Oil Red O quantities and normalised to the cell number per well using DAPI staining.

### 2.14. Statistical Analysis

All the data are presented as the mean ± standard error of mean (SEM). Normality was determined using the Shapiro–Wilk test, and the data were analysed using either unpaired *t*-tests or Mann–Whitney tests. Ordinary one-way ANOVA was used when comparing more than two groups.

## 3. Results

### 3.1. Metabolic Characterisation of AdipoQ-mCAT TG Mice

**Figure 1:**
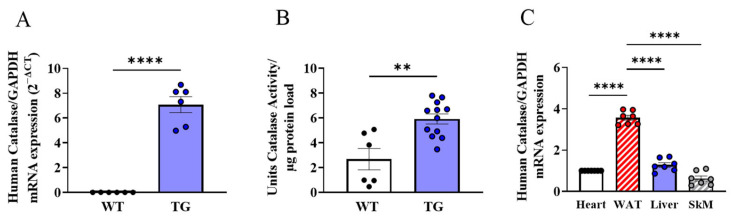
**Human catalase overexpression in white adipose tissue of AdipoQ-mCAT TG mice** (**A**). WAT mRNA expression levels of human catalase in wild-type (WT) vs. AdipoQ-mCAT transgenic (TG) mice on NC diet (*n* = 6 per group) (**B**). Overall catalase activity levels in adipose tissue of WT (*n* = 6) vs. AdipoQ-mCAT TG (*n* = 11) mice on NC diet (**C**). Human catalase mRNA expression levels in heart, WAT, liver, and skeletal muscle of AdipoQ-mCAT mice (*n* = 7). For statistical analysis, unpaired *t*-tests were performed for (**A**,**B**), and ordinary one-way ANOVA was performed in (**C**). Normality was assessed using the Shapiro–Wilk test. All values are represented as means with error bars representing SEM. ** *p* < 0.01, **** *p* < 0.0001.

The adipose tissue-targeted mitochondrial catalase overexpression murine transgenic line generated for use in these experiments was designated AdipoQ-mCAT. In these mice, human catalase was over-expressed at the mRNA level (Figure 1A, *p* < 0.0001), with a corresponding increase in the overall catalase activity levels (Figure 1B, *p* < 0.01) in the white adipose tissue (WAT) of AdipoQ-mCAT TG mice compared to the wild-types (WT). Furthermore, the human catalase mRNA was significantly up-regulated in the WAT compared to the heart (3.6-fold, ±0.12 SEM), liver (2.8-fold, ±0.11 SEM), and skeletal muscle (5.7-fold, ±0.12 SEM) in AdipoQ-mCAT mice (Figure 1C, WAT vs. other tissues, *p* < 0.0001).

**Figure 2:**
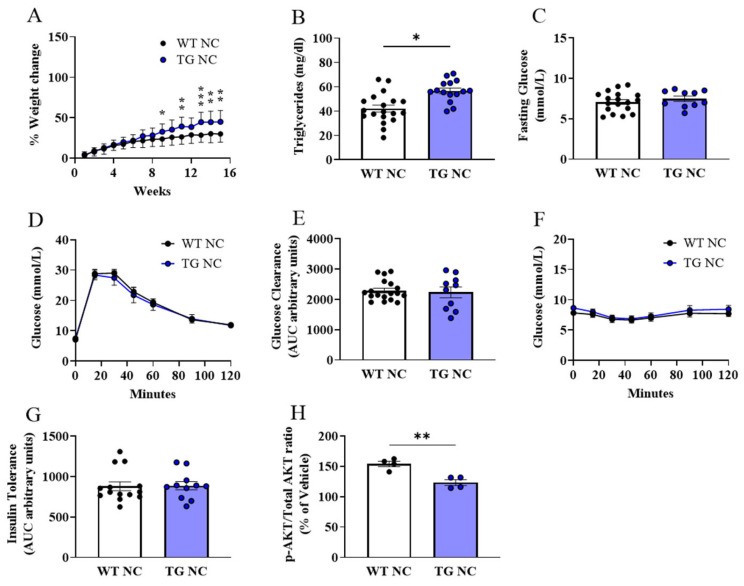
**Weight gain and metabolic parameters of WT and AdipoQ-mCAT TG mice under physiological diet conditions** (**A**). Percentage weight change over time from baseline (**B**). Plasma triglyceride levels (**C**). Fasting plasma glucose levels (**D**,**E**). Area under the curve analysis of IP-glucose tolerance test (**F**,**G**). Area under the curve analysis of IP-insulin tolerance test (**H**). Phosphorylated AKT/Total AKT ratio displayed as a percentage of vehicle control in insulin-stimulated adipose tissue. For statistical analysis, 2-way ANOVA was performed in (**A**); Mann–Whitney test was performed in (**E**,**G**); unpaired *t*-test was performed on (**B**,**C**,**H**). Normality was assessed using the Shapiro–Wilk test. All values are represented as means with error bars representing SEM. * *p* < 0.05, ** *p* < 0.01, *** *p* < 0.001.

On the normal chow (NC) diet, the AdipoQ-mCAT transgenic mice (TG) had higher body weights than those of the wild-type (WT) mice over the 16-week period, with significant differences in the per cent gain in body weight by week 9 (Figure 2A). The plasma triglyceride concentration was significantly increased in the TG mice versus the WTs (Figure 2B, *p* < 0.05). Systemic metabolic testing, including fasting blood glucose (Figure 2C) and glucose and insulin tolerance testing (Figure 2D–G), showed no difference in the responses between the groups. To investigate if the AdipoQ-mCAT TG status can alter local WAT insulin sensitivity, we measured the phosphorylated AKT/Total AKT levels in response to ex vivo insulin stimulation (Figure 2H). There was a significant reduction in the insulin-stimulated *p*-Akt/total Akt in the WAT depots of the AdipoQ-mCAT TG mice vs. the WT on a normal chow diet (*p* < 0.05), suggesting that local adipose tissue insulin responses are reduced in TG mice.

**Figure 3:**
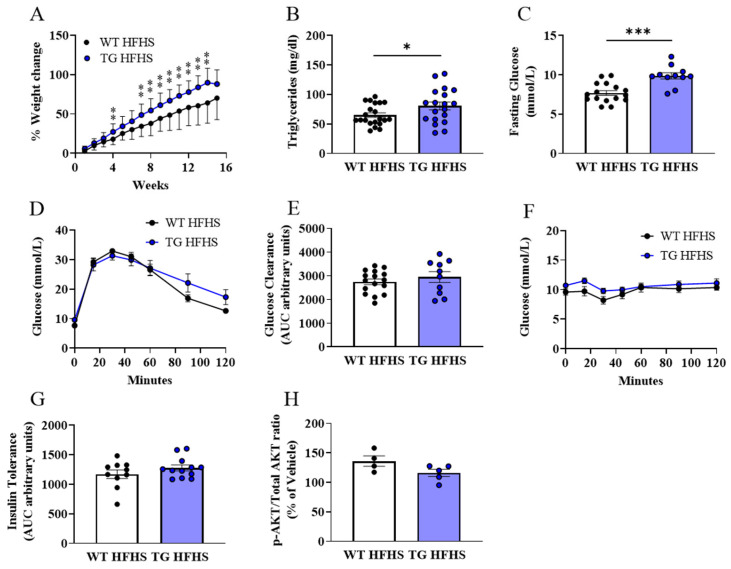
**Weight gain and metabolic parameters of WT and AdipoQ-mCAT TG mice under obesogenic stress** (**A**). Percentage weight change over time from baseline (**B**). Plasma triglyceride levels (**C**). Fasting plasma glucose levels (**D**,**E**). Area under the curve analysis of IP-glucose tolerance test (**F**,**G**). Area under the curve analysis of IP-insulin tolerance test (**H**). Phosphorylated AKT/Total AKT ratio displayed as a percentage of vehicle control in insulin-stimulated adipose tissue. For statistical analysis, 2-way ANOVA was performed in (**A**) and unpaired *t*-test was performed on (**B**,**C**,**E**,**G**,**H**). Normality was assessed using the Shapiro–Wilk test. All values are represented as means with error bars representing SEM. * *p* < 0.05, ** *p* < 0.01, *** *p* < 0.001.

To induce obesity, both the WT and AdipoQ-mCAT TG mice were given a high-fat, high-sucrose (HFHS) diet for 16 weeks. The percentage weight change was increased in the AdipoQ-mCAT vs. WT mice and was significantly different after 4 weeks of HFHS feeding (Figure 3A). As expected, the WT mice fed a HFHS diet had marked increases in triglyceride levels, impaired glucose, and insulin tolerance testing vs. the WT mice fed an NC diet (Supplementary Figure S1A–H). While the triglycerides and fasting glucose levels were significantly higher in the TG mice on the HFHS diet vs. the WT (Figure 3B,C), there was no incremental impairment of the systemic glucose and insulin responses (Figure 3D–G) or the local adipose tissue responsiveness to insulin (Figure 3H) between these two groups.

### 3.2. Characterisation of Adipose Tissue Changes in AdipoQ-mCAT TG vs. WT Mice Fed Normal Chow

**Figure 4:**
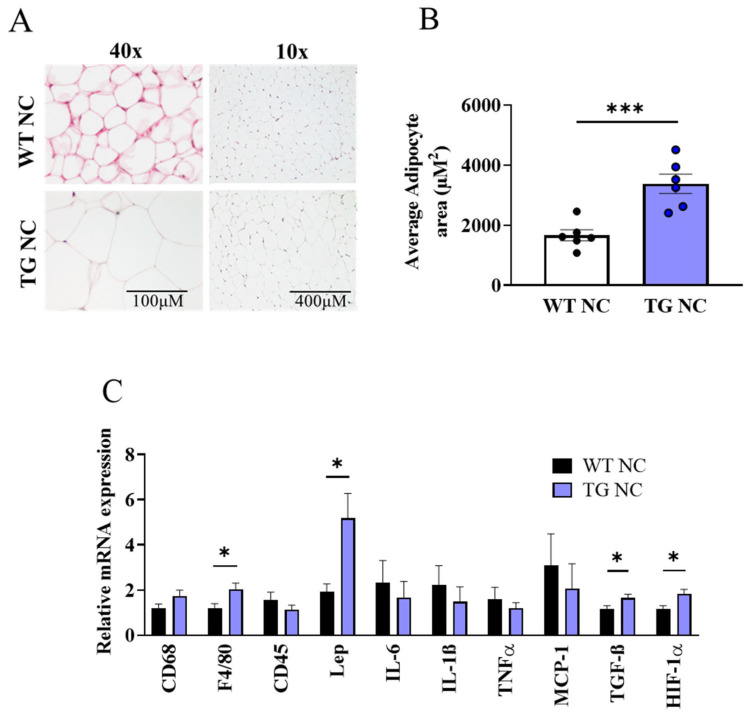
**Characterisation of white adipose tissue in AdipoQ-mCAT TG mice under physiological diet conditions** (**A**). Representative H & E staining of white adipose tissue. Left panel 40× magnification (Scale bar = 100 μM), right panel 10× magnification (Scale bar = 400 μM) (**B**). Average adipocyte area quantitated from image analysis of H & E staining (*n* = 6–7 per group) (**C**). Quantitative PCR analysis of immune cell markers, adipokines, cytokines, and hypoxia markers as indicated on graphs (*n* = 9–10 per group). For statistical analysis, unpaired *t*-test was used for (**B**), and Mann–Whitney test was used for (**C**). Normality was tested using the Shapiro–Wilk test. All values are represented as means with error bars representing SEM. * *p* < 0.05 *** *p* < 0.001.

We investigated the effect of AdipoQ-mCAT overexpression on the key indicators of adipose tissue health: morphology, adipogenic capacity, adipokine expression, and inflammatory and hypoxic state. The adipocyte area was significantly enlarged in the AdipoQ-mCAT TG mice versus the WTs on the NC diet (Figure 4A,B, *p* < 0.001). This was further demonstrated by the reduced frequency of smaller adipocytes (500–3000 μM^2^) and a shift towards larger adipocytes (3000–10,000 μM^2^) in the AdipoQ-mCAT TG mice compared to the WT mice (Supplementary Figure S2A). Characterisation of the WAT mRNA showed marked upregulation of F4/80, Leptin, TGFβ, and HIF-1α; however, CD68, CD45, IL6, IL-1β, TNFα, and MCP-1 were not differentially regulated between the WT vs. TG mice on the NC diet (Figure 4C).

**Figure 5:**
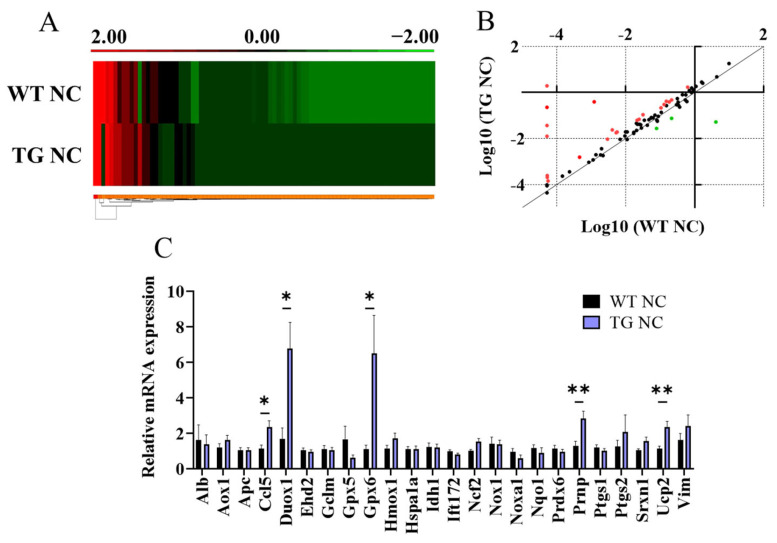
**Analysis of oxidative stress genes in adipose tissue of AdipoQ-mCAT TG mice under physiological diet conditions** (**A**). Gene expression heat map comparing oxidative stress genes in adipose tissue of WT and AdipoQ-mCAT TG mice (**B**). Scatterplot representing differentially expressed genes in an oxidative stress qPCR panel. Red: Genes over-expressed 2-fold or more in AdipoQ-mCAT TG vs. WT mice; Green: Genes under-expressed 2-fold or more in AdipoQ-mCAT TG vs. WT mice (**C**). Differentially expressed genes were validated in adipose tissue using qPCR (*n* = 6–9 per group). Normality was determined using Shapiro–Wilk test in (**C**). Unpaired *t*-test or Mann–Whitney tests were performed in (**C**). All values are represented as means with error bars representing SEM. * *p* < 0.05, ** *p* < 0.01.

To further characterise the adipose tissue of the AdipoQ-mCAT TG vs. WT mice, RT Profiler qPCR array analysis was performed on the WAT obtained from the NC-fed AdipoQ-mCAT mice vs. the WT mice. A total of 26 out of 84 genes related to oxidative stress and antioxidant defence were differentially expressed by 2-fold or more in the WAT of the AdipoQ-mCAT TG mice compared to the WT mice (Figure 5A,B, Supplementary Table S3). Of these, 23 genes had increased expression, and 3 genes had decreased expression levels. Upon validation with qRT-PCR, Ccl5 (2.1-fold, *p* < 0.05), Duox1 (4.1-fold, *p* < 0.05), Gpx6 (5.9-fold, *p* < 0.05), Prnp (2.2-fold, *p* < 0.01), and Ucp2 (2.1-fold, *p* < 0.01) were shown to have significantly increased expression in the WAT of the AdipoQ-mCAT TG mice versus the WTs (Figure 5C).

### 3.3. Obesogenic Diet-Induced Adipose Tissue Changes in AdipoQ-mCAT TG vs. WT Mice Fed HFHS Diet

**Figure 6:**
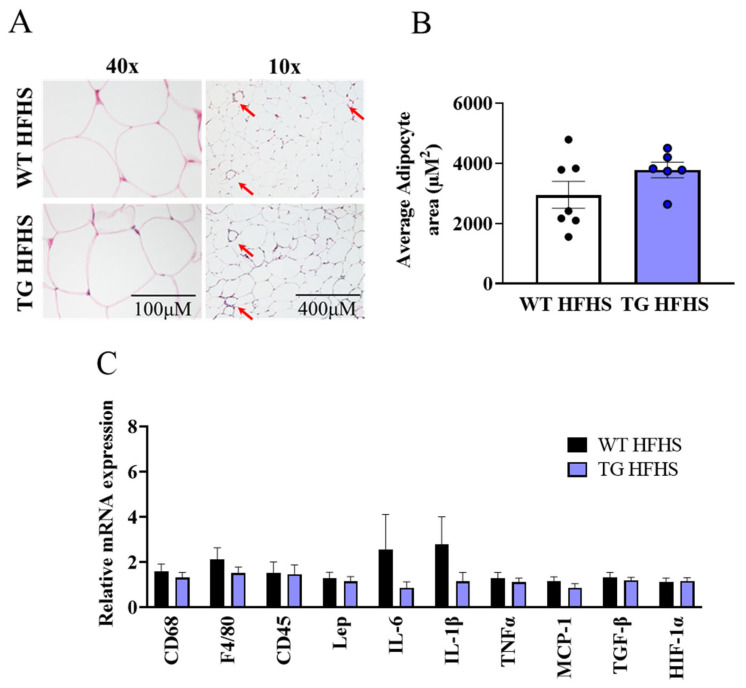
**Characterisation of white adipose tissue in AdipoQ-mCAT TG mice under obesogenic stress** (**A**). Representative H & E staining of white adipose tissue. Left panel 40× magnification (Scale bar = 100 μM), right panel 10× magnification (Scale bar = 400 μM). Crown-like structures are indicated by red arrows (**B**). Average adipocyte area quantitated from image analysis of H & E staining (*n* = 6–7 per group) (**C**). Quantitative PCR analysis of immune cell markers, adipokines, cytokines, and hypoxia markers as indicated on graphs (*n* = 9–10 per group). For statistical analysis, unpaired *t*-test was used for (**B**), and Mann–Whitney test was used for (**C**). Normality was tested using the Shapiro–Wilk test. All values are represented as means with error bars representing SEM.

The characterisation of local adipose tissue depots showed no difference in the adipocyte size (Figure 6A,B, Supplementary Figure S2B) in the AdipoQ-mCAT TG mice vs. the WT mice fed an HFHS diet. While there was marked upregulation of CD68, F4/80, Leptin, MCP-1, and HIF-1α in the WT mice on the NC diet vs. the HFHS diet (Supplementary Figure S3), there were no differences in the adipose tissue expression of the adipokine leptin, cytokines IL6, IL1β, TNFα, MCP-1, and TGFβ and hypoxia marker HIF1α between the WT and AdipoQ-mCAT TG mice on the HFHS diet (Figure 6C).

**Figure 7:**
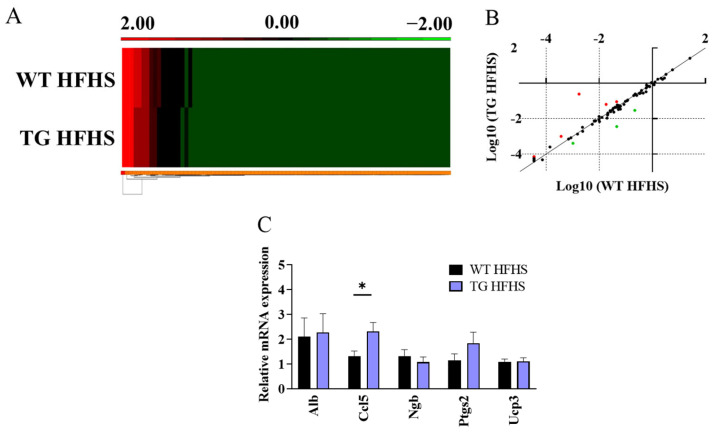
**Analysis of oxidative stress genes in adipose tissue of AdipoQ-mCAT TG mice under conditions of obesogenic stress** (**A**). Gene expression heat map comparing oxidative stress genes in adipose tissue of WT and AdipoQ-mCAT TG mice after 16 weeks of HFHS diet (**B**). Scatterplot representing differentially expressed genes in an oxidative stress qPCR panel. Red: Genes over-expressed 2-fold or more in AdipoQ-mCAT TG vs. WT mice; Green: Genes under-expressed 2-fold or more in AdipoQ-mCAT TG vs. WT mice (**C**). Differentially expressed genes were validated in adipose tissue using qPCR (*n* = 6–9 per group). Normality was determined using Shapiro–Wilk test in (**C**). Unpaired *t*-tests or Mann–Whitney tests were performed in (**C**). All values are represented as means with error bars representing SEM. * *p* < 0.05.

An analysis of differentially expressed genes related to oxidative stress and antioxidant defence revealed that while the HFHS diet caused a 2-fold or more change in 28 out of 84 genes in the WT mice (vs. the NC diet) (Supplementary Figure S4, Supplementary Table S4), only 9 out of 84 genes were incrementally changed in the HFHS-fed AdipoQ-mCAT mice versus the HFHS-fed WT mice (Figure 7A,B, Supplementary Table S5). Of these, six were up-regulated and three were down-regulated. Validation by qRT-PCR confirmed that Ccl5 was significantly increased (1.8-fold, *p* < 0.05), whereas the other genes tested were either undetectable or not significantly changed (Figure 7C).

### 3.4. Adipogenic Profiling

**Figure 8:**
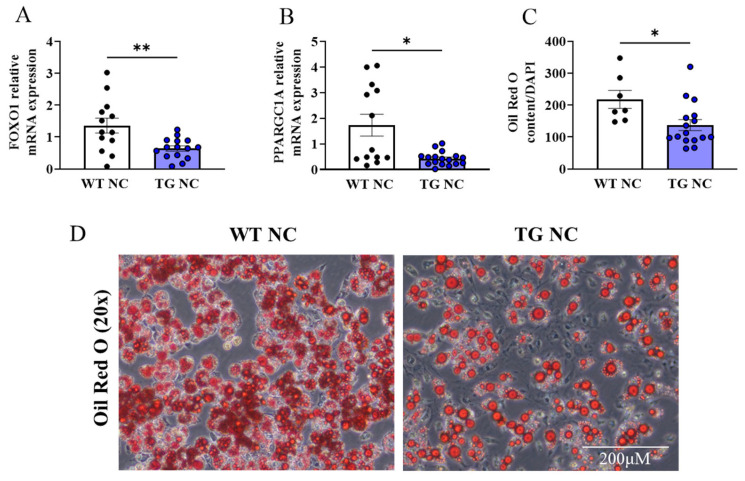
**Adipogenic profiling of AdipoQ-mCAT TG mice under physiological diet conditions.** QPCR analysis of FOXO1 (**A**) and PPARGC1A (**B**) under physiological diet conditions (**C**). Quantitation of Oil Red O staining normalised by cell number assessed by DAPI staining (**D**). Representative images of Oil Red O staining of differentiated bone marrow cells, at 20× magnification (Scale bar = 200 μM). Unpaired *t*-test or Mann–Whitney tests were performed. All values are represented as means with error bars representing SEM. * *p* < 0.05, ** *p* < 0.01.

**Figure 9:**
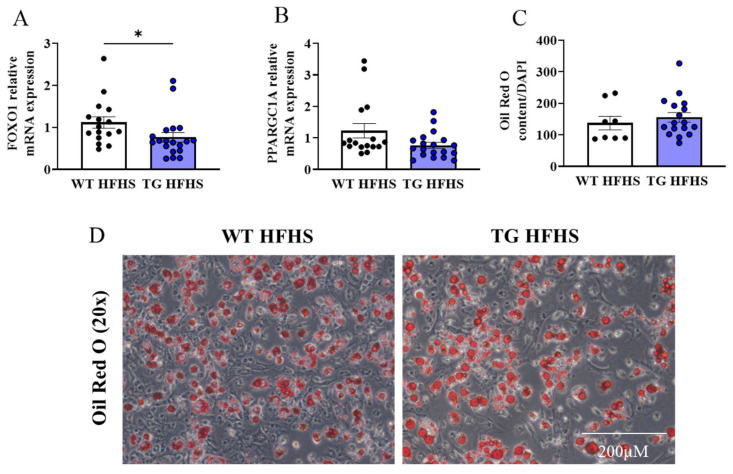
**Adipogenic profiling of AdipoQ-mCAT TG mice under HFHS diet conditions.** qPCR analysis of FOXO1 (**A**) and PPARGC1A (**B**) under HFHS diet conditions (**C**). Quantitation of Oil Red O staining normalised by cell number assessed by DAPI staining (**D**). Representative images of Oil Red O staining of differentiated bone marrow cells, at 20× magnification (Scale bar = 200 μM). Unpaired *t*-test or Mann–Whitney tests were performed. All values are represented as means with error bars representing SEM. * *p* < 0.05.

An analysis of the FOXO1 and PPARGC1A genes, shown to regulate adipogenesis [24,25], within the WAT depot, showed a significant reduction in the TG vs. WT mice fed the NC diet (Figure 8A,B). The bone marrow adipogenesis assays showed a reduction in the adipogenic capacity in the TG mice compared to the WTs under normal diet conditions (Figure 8C,D). In the HFHS-fed mice, FOXO1 mRNA expression was reduced in the WAT in the WT vs. TG mice, while PPARGC1A was unchanged (Figure 9A,B). The WT and TG mice under HFHS diet conditions showed no difference in the adipogenic capacity (Figure 9C,D).

## 4. Discussion

Increased oxidative stress and inflammation are hallmark features of adipose tissue dysregulation resulting in obesogenic stress-induced metabolic perturbations [10,26]. Strategies to specifically target adipose tissue, with the aim of reducing adipose tissue inflammation and oxidative stress, have been successful in improving systemic metabolic function in mice [27,28,29]. However, several clinical studies have shown that diet-mediated weight loss alone was not associated with long-term improvement in systemic metabolic function in human trials [30,31]. Furthermore, dietary supplementation with antioxidants has been largely unsuccessful in improving type-II diabetes risk [14,15]. Previously, we showed that impaired mitochondrial redox is associated with visceral adiposity in patients with obesity, which was linked to both local and systemic metabolic perturbations [32]. These results led us to hypothesise that increasing antioxidants within adipose tissue could confer metabolic protection. In the current study, we developed a novel mouse model overexpressing mitochondrial catalase in mature adipocytes.

Previous murine studies demonstrated that whole-body mCAT overexpression could improve high-fat diet (1–12 weeks)-induced metabolic dysfunction [33,34,35]. Similarly, studies using the pharmacological mitochondria-targeted antioxidants MitoQ or MitoTEMPO have shown improvements in fasting blood glucose, insulin, lipid levels, and glucose and insulin tolerance in rodent models of high-fat diet (7–8 weeks)-induced obesity [36,37,38]. In a longer-term model (24 weeks), MitoQ treatment was effective against impaired glucose tolerance induced by a high-fat diet [39]. While these data support that increasing mitochondrial antioxidants is protective against metabolic perturbations in obese models, studies using genetic manipulation of other mitochondrial antioxidants have yielded discordant results. Interestingly, adipocyte-specific knockout of an antioxidant enzyme, manganese superoxide dismutase (MnSOD), using AdipoQ-Cre mice was protective against high-fat diet-induced weight gain and metabolic perturbations [27]. It was postulated that the deletion of MnSOD may have triggered several stress response mechanisms to protect the mice from diet-induced metabolic dysfunction [27]. Manipulations of the potent antioxidant enzyme glutathione peroxidase (Gpx), which has a greater affinity for H_2_O_2_ than catalase [40], have yielded similar results. Gpx1 knockout (Gpx1−/−) with associated increased ROS production led to enhanced insulin signalling and protected mice from high-fat diet-induced obesity and metabolic impairment [41]. In line with these findings, increased adipose H_2_O_2_ levels in mice were found to be associated with reduced adipocyte size.Furthermore, the adipose-targeted overexpression of the antioxidant enzymes SOD1/Cat induced hypertrophy and enhanced insulin sensitivity [42].

Our results showed that without HFHS stimulation, the AdipoQ-mCAT TG mice had greater weight gain, beginning at 9 weeks of age, with associated significant increases in the adipocyte size. Our results are in line with a previous study that showed a trend towards larger adipocytes and weight gain in mice with whole-body mCAT overexpression [43]. However, we also observed accumulated plasma triglyceride levels and reduced insulin signalling in local adipose depots, suggesting that the AdipoQ-mCAT TG mice developed adipocyte remodelling.

Physiological levels of ROS are important for normal cellular functioning. Hydrogen peroxide, while implicated in oxidative stress and cellular damage, is also known to function as a signalling molecule in physiological processes, including insulin signalling [44]. Of the reactive oxygen species, H_2_O_2_ is relatively stable, long-lived, and can pass through biological membranes, making it an ideal signalling molecule [45,46]. In adipocytes, while exogenous H_2_O_2_ treatment in 3T3-L1 cells led to insulin resistance, NOX4-derived H_2_O_2_ has been shown to maintain physiological insulin signalling in cultured adipocytes [47].

Interestingly, our results showed that while HFHS diet-induced obesity induced marked weight gain, triglycerides accumulation, and the systemic impairment of glucose and insulin in the WT mice vs. those fed the NC diet, the AdipoQ-mCAT TG mice fed the HFHS diet were protected from incremental metabolic impairment compared to the WT mice. In a previous study, a physiological increase in cardiac catalase activity levels in mitochondria due to obesity was able to limit H_2_O_2_ levels while maintaining normal insulin responsiveness in cardiac tissue [40]. However, a 50-fold increase in catalase activity with mitochondrial catalase overexpression completely blocked insulin responsiveness [40].

In our model, the increase in catalase activity was associated with an increased adipocyte size, reduced adipose tissue insulin responses, and significant gene changes associated with the adipose remodelling in the AdipoQ-mCAT TG vs. WT mice, in the absence of obesogenic stress induced by HFHS feeding. We also observed lower WAT mRNA FOXO1 and PPARGC1A gene expression, with an associated reduction in adipogenic capacity, in the AdipoQ-mCAT TG vs. WT mice fed an NC diet. Reactive oxygen species and adipogenic differentiation are inter-regulated. Several studies have demonstrated that ROS promote adipogenesis [48,49] and that FOXO1 transcription plays an important role in maintaining cellular redox homeostasis during the adipogenic process [50,51]. Consistent with the literature, our results showed a reduction in adipogenesis in AdipoQ-mCAT, suggesting that ROS scavenging can inhibit adipogenesis [52] and concomitant FOXO1 inhibition.

However, with obesogenic stress induced by HFHS feeding, our results showed that the increase in catalase activity in the AdipoQ-mCAT TG mice did not result in any improvement in the systemic metabolic profiles or adipogenic changes. Our exploratory examination of WAT gene profiling has demonstrated a number of local expression changes in a panel of oxidative stress-related genes consistent with adipose tissue remodelling. Under normal (physiological) feeding conditions, there was marked upregulation of inflammatory markers, such as Ccl5, macrophage marker F4/80, TGF-β, and HIF-1α; these changes were abrogated in the presence of obesogenic stress with HFHS feeding in the AdipoQ-mCAT TG vs. WT mice. Physiologically, ROS are critically important for redox-sensitive signalling pathways that modify multiple regulatory proteins that are critical in cellular homeostasis and involved in the control of pro-inflammatory, pro-fibrotic signalling, cell proliferation, and apoptosis [45,46,53,54]. Consistent with the literature, we observed marked upregulation of oxidative gene changes in the WT mice fed an HFHS diet vs. an NC diet [55,56,57]. However, there was no differential upregulation of oxidative genes in HFHS feeding with the AdipoQ-mCAT TG mice vs. the NC diet. We speculate that the AdipoQ-mCAT modification of catalase-mediated ROS could have negative feedback activation of cellular defence and inflammatory and fibrotic systems under normal physiological conditions. The lack of the same adipose gene changes with HFHS in these mice suggests that there is potentially catalase-mediated protection against H_2_O_2_-mediated adipose tissue damage to accommodate for WAT expansion with HFHS feeding.

One of the limitations of our study is that our study is largely descriptive, as we did not explore all the oxidative changes behind the different effects of catalase overexpression in relation to systemic metabolic activity. An evaluation of the effects of shorter and longer-term HFHS feeding would need to be undertaken to fully elucidate the mechanisms related to catalase-mediated effects on metabolic status. Given the fact that visceral adiposity is most commonly associated with adverse metabolic profiles, we concentrated on visceral WAT in this model. We have yet to confirm the effects of catalase-mediated adipocyte-specific oxidative phosphorylation capacity in relation to obesogenic stress-induced metabolic dysfunction. While we did not measure ROS production in this model, we and others [18,21,22,58,59] have extensively demonstrated that the overexpression of mitochondrial catalase is associated with a marked reduction in H_2_O_2_ production and an associated reduction of tissue-specific protein cysteine modifications.

In summary, our study demonstrates that adipose-targeted mCAT expression can result in adipocyte redox imbalance under normal diet conditions to the detriment of metabolic parameters and the adipose tissue microenvironment. However, during HFHS diet-induced obesity, where oxidative stress levels are higher, while mCAT expression in adipose tissue could not improve metabolic perturbations or the adipose tissue environment, it did prevent further exacerbation of obesity-induced metabolic impairment. We showed that the overexpression of mitochondrial catalase within adipose tissue alone was not sufficient to confer systemic metabolic protection against diet-induced obesity. Our results highlight that obesity is a complex and multifactorial disease beyond adipose tissue dysregulation.

These results highlight the critical role of physiological H_2_O_2_ signalling in metabolism and the adipose tissue environment and the need for further studies on the effects of antioxidants during HFHS diet-induced obesity.

## Data Availability

The data presented in this study are available in the current article and Supplementary Materials.

## Supplementary Materials

The following supporting information can be downloaded at https://www.mdpi.com/article/10.3390/antiox12051137/s1, Supplementary Table S1: Pre-designed qPCR primers; Supplementary Table S2: qPCR primer sequences; Supplementary Table S3: Differentially expressed (>2-fold) oxidative stress genes in adipose tissue of AdipoQ-mCAT mice versus Wild-type; Supplementary Table S4: Differentially expressed (>2-fold) oxidative stress genes in adipose tissue of Wild-type mice fed NC vs. HFHS diet; Supplementary Table S5: Differentially expressed (>2-fold) oxidative stress genes in adipose tissue of AdipoQ-mCAT HFHS mice versus Wild-type HFHS; Supplementary Figure S1: Weight gain and metabolic parameters of WT mice under obesogenic stress; Supplementary Figure S2: Adipocyte size frequency histogram; Supplementary Figure S3: Gene expression analysis of white adipose tissue of WT mice under obesogenic stress; Supplementary Figure S4: Analysis of oxidative stress genes in adipose tissue of WT mice under conditions of obesogenic stress.
