# Supplementary material for: Overexpression of Mitochondrial Catalase within Adipose Tissue Does Not Confer Systemic Metabolic Protection against Diet-Induced Obesity
Source: Antioxidants (Basel). 2023 May 22;12(5):1137. doi: 10.3390/antiox12051137 (PMC10215245; doi:10.3390/antiox12051137)
Supplement: Supplementary file 1 [file antioxidants-12-01137-s001.zip › antioxidants-2209361-supplementary.pdf]

antioxidants MDPI

Supplementary Materials

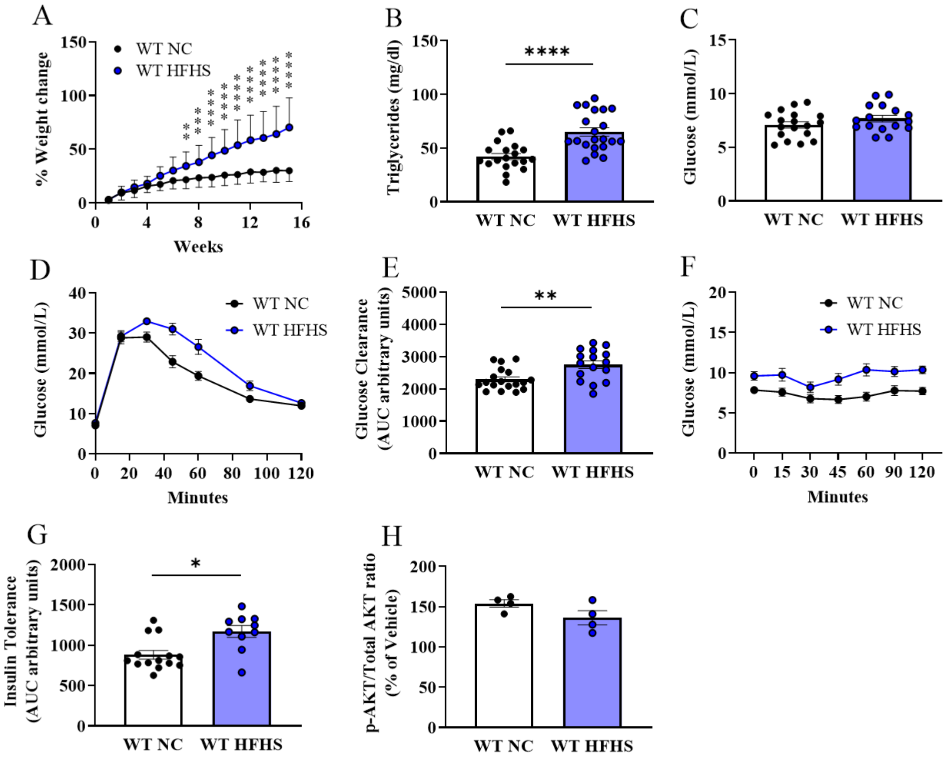

Figure S1. Weight gain and metabolic parameters of WT mice under obesogenic stress. A. Percentage weight change at endpoint from baseline. B. Plasma triglyceride levels. C. Fasting plasma glucose levels. D-E. Area under the curve analysis of IP-glucose tolerance test. F-G. Area under the curve analysis of IP-insulin tolerance test. H. Phosphorylated AKT/Total AKT ratio displayed as a percentage of vehicle control in insulin-stimulated adipose tissue. For statistical analysis, 2-way ANOVA was performed in A; Mann-Whitney test was performed in E and G; unpaired t-test was performed on B, C and H. Normality was assessed using the Shapiro-Wilk test. All values are represented as means with error bars representing SEM. * $p < 0.05$, ** $p < 0.01$, **** $p < 0.0001$.

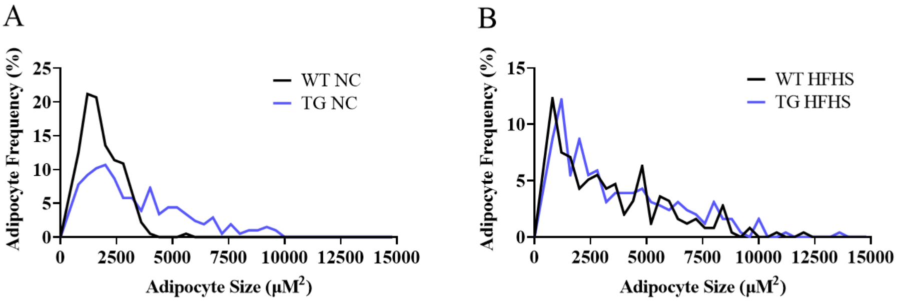

Figure S2. Adipocyte size frequency histogram. A. Frequency histogram of adipocytes by area (μM²) in WT vs AdipoQ-mCAT TG mice under physiological diet conditions. B. Frequency histogram of adipocytes by area (μM²) in WT vs AdipoQ-mCAT mice under obesogenic stress.

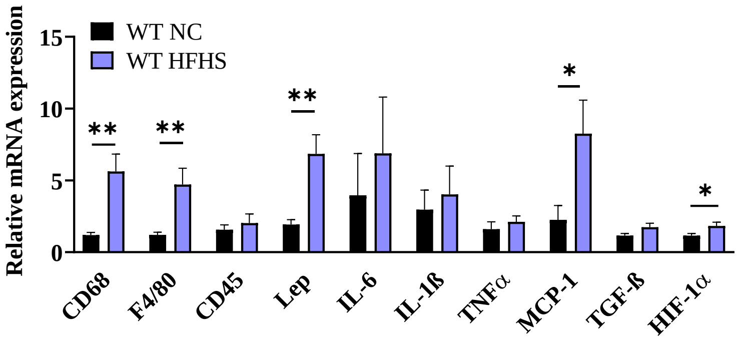

Figure S3. Gene expression analysis of white adipose tissue of WT mice under obesogenic stress. A. Quantitative PCR analysis of gene expression in white adipose tissue of WT mice fed NC diet versus HFHS diet (n = 9,10 per group). For statistical analysis, unpaired t-tests or Mann-Whitney tests were performed. Normality was assessed using the Shapiro-Wilk test. All values are represented as means with error bars representing SEM. * $p < 0.05$, ** $p < 0.01$.

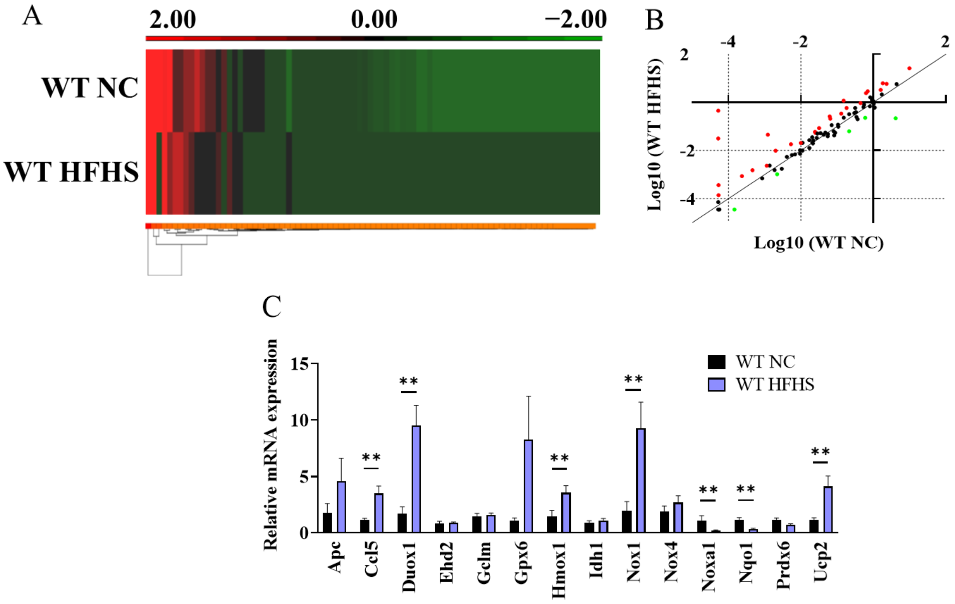

Figure S4. Analysis of oxidative stress genes in adipose tissue of WT mice under conditions of obesogenic stress. A. Gene expression heat map comparing oxidative stress genes in adipose tissue of WT mice after 16 weeks of HFHS diet compared to NC diet. B. Scatterplot representing differentially expressed genes in an oxidative stress qPCR panel. Red: Genes over-expressed 2-fold or more in WT HFHS vs WT NC mice; Green: Genes under-expressed 2-fold or more in WT HFHS vs WT NC mice. C. Differentially expressed genes were validated in adipose tissue using qPCR (n = 6–9 per group). Normality was determined using Shapiro-Wilk test in B-D. Unpaired t-tests or Mann-Whitney tests were performed in C. All values are represented as means with error bars representing SEM. ** $p < 0.01$.

Table S1. Pre-designed qPCR primers.

Primer Name	Catalogue Number	Manufacturer
Mouse IL6	M_Il6_1	Sigma-Aldrich KicqStart Primers
Mouse IL1B	M_Il1b_1	Sigma-Aldrich KicqStart Primers
Mouse LEP	M_Lep_1	Sigma-Aldrich KicqStart Primers
Mouse MCP-1	M_Ccl2_1	Sigma-Aldrich KicqStart Primers
Mouse HIF1a	M_Hif1a_1	Sigma-Aldrich KicqStart Primers
Mouse Duox1	M_Duox1_1	Sigma-Aldrich KicqStart Primers
Mouse Gpx5	M_Gpx5_1	Sigma-Aldrich KicqStart Primers

Mouse Noxa1	M_Noxa1_1	Sigma-Aldrich KicqStart Primers
Mouse Nqo1	M_Nqo1_1	Sigma-Aldrich KicqStart Primers
Mouse Prdx6	M_Prdx6_1	Sigma-Aldrich KicqStart Primers
Human Catalase	Hs00156308_m1	Thermo Fisher Taqman Assay
Mouse GAPDH	4326317	Thermo Fisher Taqman Assay

Table S2. qPCR primer sequences.

Primer Name	Forward Primer 5' -> 3'	Reverse Primer 5' -> 3'
Mouse CD68	AGGACCGCTTATAGCCCAAG	GGATGGCAGGAGAGTAACGG
Mouse F4/80	GTGACTCACCTTGTGGTCCT	CAGACACTCATCAACATCTGCG
Mouse CD45	AGAAGTCTTTGTCACAGGGCA	AAGAAGGGCATTCTCTGTTGTG
Mouse TGFB1	ACTGGAGTTGTACGGCAGTG	GGGGCTGATCCCGTTGATT
Mouse TNFA	CCCACGTCGTAGCAAACCA	ACAAGGTACAACCCATCGGC
Mouse PPIA	CATTCCTGGACCCAAAACG	GGCAAATGCTGGACCAAAC
Mouse Ccl5	ATATGGCTCGGACACCACTC	CTTCGAGTGACAAACACGACTG
Mouse Gclm	ACAATGACCCGAAAGAACTGC	GAGCTGGAGTTAAGAGCCCC
Mouse Gpx6	GTCACGGTTTTGGGCTTTCC	GCCTGGACGCACATACTTGA
Mouse Hmox1	GCCCCACCAAGTTCAAACAG	GCTCCTCAAACAGCTCAATGT
Mouse Nox1	GTTTCTCTCCCGAAGGACCTCT	GGCCAAGGCAGTCCCAAGAA
Mouse Nox4	CCAAATGTTGGGCGATTGTGT	AGGATGAGGCTGCAGTTGAG
Mouse Apc	CCCTATGTACGCCTTCCTGC	CTACCCTTGGACCTGCCAGA
Mouse Ehd2	TCGTTGTGGACCCAGAGAAG	GGCGCACATAAACCTGTTG
Mouse Idh1	TTCCGAGCGAAGGTTGTGG	TTTTACTTCAATAACCCGGCTCAC
Mouse Ucp2	CCTCCCTTGCCACTTCACTT	GGAAGGCATGAACCCCTTGTA
Mouse Hsp90ab1	AGATTCCACTAACCGACGCC	TGCTCTTTGCTCTCACCAGT
Mouse GAPDH	TACGGGTGCACGTAGCTCA	AATACGGCCAAATCCGTTCAC

Table S3. Differentially expressed (>2-fold) oxidative stress genes in adipose tissue of AdipoQ-mCAT mice versus Wild-type.

Gene Symbol	Refseq ID	Gene Name	Fold Regulation (vs Wild-type)
Alb	NM_009654	Albumin	−79.35
Aox1	NM_009676	Aldehyde oxidase 1	2.78
Apc	NM_007462	Adenomatosis polyposis coli	2.25
Ccl5	NM_013653	Chemokine (C-C motif) ligand 5	3.37
Duox1	NM_001099297	Dual oxidase 1	3.36
Ehd2	NM_153068	EH-domain containing 2	2.13
Gclm	NM_008129	Glutamate-cysteine ligase, modifier subunit	2.03
Gpx5	NM_010343	Glutathione peroxidase 5	37222.98
Gpx6	NM_145451	Glutathione peroxidase 6	241.36
Hmox1	NM_010442	Heme oxygenase (decycling) 1	−2.84
Hspa1a	NM_010479	Heat shock protein 1A	3.41
Idh1	NM_010497	Isocitrate dehydrogenase 1 (NADP+), soluble	4354.68
Ift172	NM_026298	Intraflagellar transport 172 homolog (Chlamydomonas)	708.21
Mb	NM_013593	Myoglobin	3.17
Mpo	NM_010824	Myeloperoxidase	3.87
Ncf2	NM_010877	Neutrophil cytosolic factor 2	3.41
Nox1	NM_172203	NADPH oxidase 1	4.68
Noxa1	NM_172204	NADPH oxidase activator 1	2.57

Nqo1	NM_008706	NAD(P)H dehydrogenase, quinone 1	5.85
Prdx6	NM_007453	Peroxiredoxin 6	2.22
Prnp	NM_011170	Prion protein	2.31
Ptgs1	NM_008969	Prostaglandin-endoperoxide synthase 1	2.89
Ptgs2	NM_011198	Prostaglandin-endoperoxide synthase 2	319.82
Srxn1	NM_029688	Sulfiredoxin 1 homolog (S. cerevisiae)	−2.84
Ucp2	NM_011671	Uncoupling protein 2 (mitochondrial, proton carrier)	2.72
Vim	NM_011701	Vimentin	2.72

Table S4. Differentially expressed (>2-fold) oxidative stress genes in adipose tissue of Wild-type mice fed NC vs HFHS diet.

Gene Symbol	Refseq ID	Gene Name	Fold Regulation (vs Wild-type NC)
Alb	NM_009654	Albumin	−18.87
Aox1	NM_009676	Aldehyde oxidase 1	2.43
Ccl5	NM_013653	Chemokine (C-C motif) ligand 5	3.41
Ctsb	NM_007798	Cathepsin B	4.08
Cyba	NM_007806	Cytochrome b-245, alpha polypeptide	4.14
Cygb	NM_030206	Cytoglobin	2.13
Duox1	NM_001099297	Dual oxidase 1	3.28
Ehd2	NM_153068	EH-domain containing 2	3.18
Epx	NM_007946	Eosinophil peroxidase	−4.21
Gpx1	NM_008160	Glutathione peroxidase 1	2.04
Gpx7	NM_024198	Glutathione peroxidase 7	2.06
Gstp1	NM_013541	Glutathione S-transferase, pi 1	−2.64
Idh1	NM_010497	Isocitrate dehydrogenase 1 (NADP+), soluble	8689.38
Ift172	NM_026298	Intraflagellar transport 172 homolog (Chlamydomonas)	604.75
Mpo	NM_010824	Myeloperoxidase	6.84
Ncf2	NM_010877	Neutrophil cytosolic factor 2	2.68
Ngb	NM_022414	Neuroglobin	−2.17
Nox1	NM_172203	NADPH oxidase 1	2.64
Nox4	NM_001285833	NADPH oxidase 4	4.86
Prnp	NM_011170	Prion protein	2.61
Ptgs2	NM_011198	Prostaglandin-endoperoxide synthase 2	37.03
Recql4	NM_058214	RecQ protein-like 4	3.65
Scd1	NM_009127	Stearoyl-Coenzyme A desaturase 1	2.62
Serpinb1b	NM_173052	Serine (or cysteine) peptidase inhibitor, clade B, member 1b	2.05
Sod3	NM_011435	Superoxide dismutase 3, extracellular	2.09
Srxn1	NM_029688	Sulfiredoxin 1 homolog (S. cerevisiae)	−3.43
Ucp2	NM_011671	Uncoupling protein 2 (mitochondrial, proton carrier)	7.72
Vim	NM_011701	Vimentin	3.85

Table S5. Differentially expressed (>2-fold) oxidative stress genes in adipose tissue of AdipoQ-mCAT HFHS mice versus Wild-type HFHS.

Gene Symbol	Refseq ID	Gene Name	Fold Regulation (vs Wild-type)
Ccl5	NM_013653	Chemokine (C-C motif) ligand 5	3.55

Il19	NM_001009940	Interleukin 19	2.01
Lpo	NM_080420	Lactoperoxidase	2.05
Mb	NM_013593	Myoglobin	140.79
Mpo	NM_010824	Myeloperoxidase	2.73
Ucp3	NM_009464	Uncoupling protein 3 (mitochondrial, proton carrier)	2.03
Alb	NM_009654	Albumin	−7.39
Ngb	NM_022414	Neuroglobin	−2.53
Ptgs2	NM_011198	Prostaglandin-endoperoxide synthase 2	−12.67
